# Deletion of *Sema3a* or *plexinA1/plexinA3* Causes Defects in Sensory Afferent Projections of Statoacoustic Ganglion Neurons

**DOI:** 10.1371/journal.pone.0072512

**Published:** 2013-08-26

**Authors:** Kei-ichi Katayama, Fumiyasu Imai, Fumikazu Suto, Yutaka Yoshida

**Affiliations:** 1 Division of Developmental Biology, Cincinnati Children’s Hospital Medical Center, Cincinnati, Ohio, United States of America; 2 Department of Ultrastructural Research, National Institute of Neuroscience, National Center of Neurology and Psychiatry, Kodaira, Tokyo, Japan; School of Biomedical Sciences, The University of Queensland, Australia

## Abstract

Statoacoustic ganglion (SAG) neurons project sensory afferents to appropriate targets in the inner ear to form functional vestibular and auditory circuits. Neuropilin1 (Npn1), a receptor for class 3 semaphorins, is required to generate appropriate afferent projections in SAG neurons; however, the ligands and coreceptors involved in Npn1 functioning remain unknown. Here we show that both *plexinA1* and *plexinA3* are expressed by SAG neurons, and *plexinA1/plexinA3* double mutant mice show defects in afferent projections of SAG neurons in the inner ear. In control mice, sensory afferents of SAG neurons terminate at the vestibular sensory patches, whereas in *plexinA1/plexinA3* double mutants, they extend more dorsally in the inner ear beyond normal vestibular target areas. Moreover, we find that *semaphorin3a* (*Sema3a)* is expressed in the dorsal otocyst, and *Sema3a* mutant mice show defects in afferent projections of SAG neurons similar to those observed in *plexinA1/plexinA3* double mutants and in mice lacking a functional Npn1 receptor. Taken together, these genetic findings demonstrate that Sema3a repellent signaling plays a role in the establishment of proper afferent projections in SAG neurons, and this signaling likely occurs through a receptor complex involving Npn1 and either plexinA1 or plexinA3.

## Introduction

The mammalian inner ear contains a group of mechanosensitive sensory organs that mediate hearing and balance. The vestibular sensory organs, including cristae in the three semicircular canals and maculae in the saccule and utricle, detect acceleration and gravity, while the organ of Corti in the cochlea is specialized for hearing. During development, neuroblasts delaminate from the otocyst and migrate to form the statoacoustic ganglion (SAG). SAG neurons then send peripheral processes to sensory patches and gradually innervate mechanosensory hair cells in order to transmit vestibular and auditory information to the brain. Multiple guidance cues appear to be involved in the navigation of SAG neurons [1]–[6], however, compared to other types of sensory neurons, much less is known about the molecular mechanisms directing the sensory afferent projections of SAG neurons in the inner ear.

Semaphorins, which include secreted, transmembrane and GPI-anchored proteins, comprise one of the largest known families of guidance molecules [7]–[10]. Among them are class 3 semaphorins which are secreted proteins that mediate signal transduction mainly by forming complexes with Npns and plexinAs. In these complexes, Npns serve as the ligand binding components while plexins act as the signal transducers [7]–[10]. Gu *et al.* generated a knock-in mouse line which expresses a variant of Npn1 that fails to bind semaphorins (*Npn1* ^Sema-^) [11]. In these mutants, some sensory afferents of SAG neurons extend beyond their sensory targets and project dorsally to reach the skin [11]. However, no specific ligands or coreceptors for Npn1 have been identified that influence the afferent projections of SAG neurons in the developing inner ear.

Previous studies have shown that *semaporin3e* (*Sema3e*) is expressed in the dorsal otocyst [12] and while *plexinA1* and *plexinA3* are expressed in SAG neurons [13]. These three proteins are ideally localized to interact with Npn1 and participate in sculpting developing neural circuits [3]. In the present study, we examined the expression of all *plexinAs* and *Sema3s* in the inner ear. We found defects in afferent projections of SAG neurons in *plexinA1*/*plexinA3* double and *Sema3a* single mutant mice. Our genetic findings, together with previous results [11], indicate that sensory afferent projections of SAG neurons are constrained within their appropriate target regions by Sema3a-Npn1 signaling, and that this signaling likely occurs via a complex with plexinA1 or plexinA3 coreceptors.

## Materials and Methods

### Animals

The following mouse strains were used in this study: *plexinA1* mutants [14], *plexinA3* mutants [15], *Sema3a* mutants [16], and *Sema3e* mutants [17]. Mouse handling and other procedures were approved by the Institutional Animal Care and Use Committee at the Cincinnati Children’s Hospital Research Foundation.

### Morphological Analysis

For histological analyses, embryos were fixed overnight in 4% paraformaldehyde, cryoprotected in 30% sucrose, and sectioned at 16 µm on a cryostat. *In situ* hybridization analysis was performed on sections obtained from wild-type embryos as described previously [18], [19]. The following primers were used to generate probes: plexinA2 5′-CTGCATGCTACAGAGTTCAATATGC-3′ and 5′-GGATGGACAGGGAGGATTTATG-3′, Sema3a 5′-CGACAAGATATAAGGAATGGAGACC-3′ and 5′-CTCCAGTACATACAAACACGAGTG-3′, Sema3b 5′-CCCAAGTTTGTCAAGGTCTTTTGG-3′ and 5′-GAGAAGAGTCTCCAGAGCATAG-3′, and Sema3f 5′-CACAGGATTACATCTTCTACCTGG-3′ and 5′-CTTAACAGGTGCTGGTTCCTTG-3′. For other genes, we used EST clones (Invitrogen) to produce probes [14]. Nissl staining was also performed as described previously [18], [19]. To reveal the general innervation patterns of the inner ear, nerve fibers were visualized with the lipophilic tracer, DiI (Invitrogen). DiI crystals were placed into the brainstem alar plate of embryonic day 13.5 (E13.5) embryos to label eighth nerve afferent fibers to the inner ear [20]. After a diffusion time of 3 to 4 days at 37°C, inner ears were then dissected and viewed as whole-mount surface preparations. Images were taken with a BX51 microscope (Olympus).

## Results

### Expression Patterns of Npn1, plexinAs and Class 3 Semaphorins in the Developing Inner Ear

**Figure 1:**
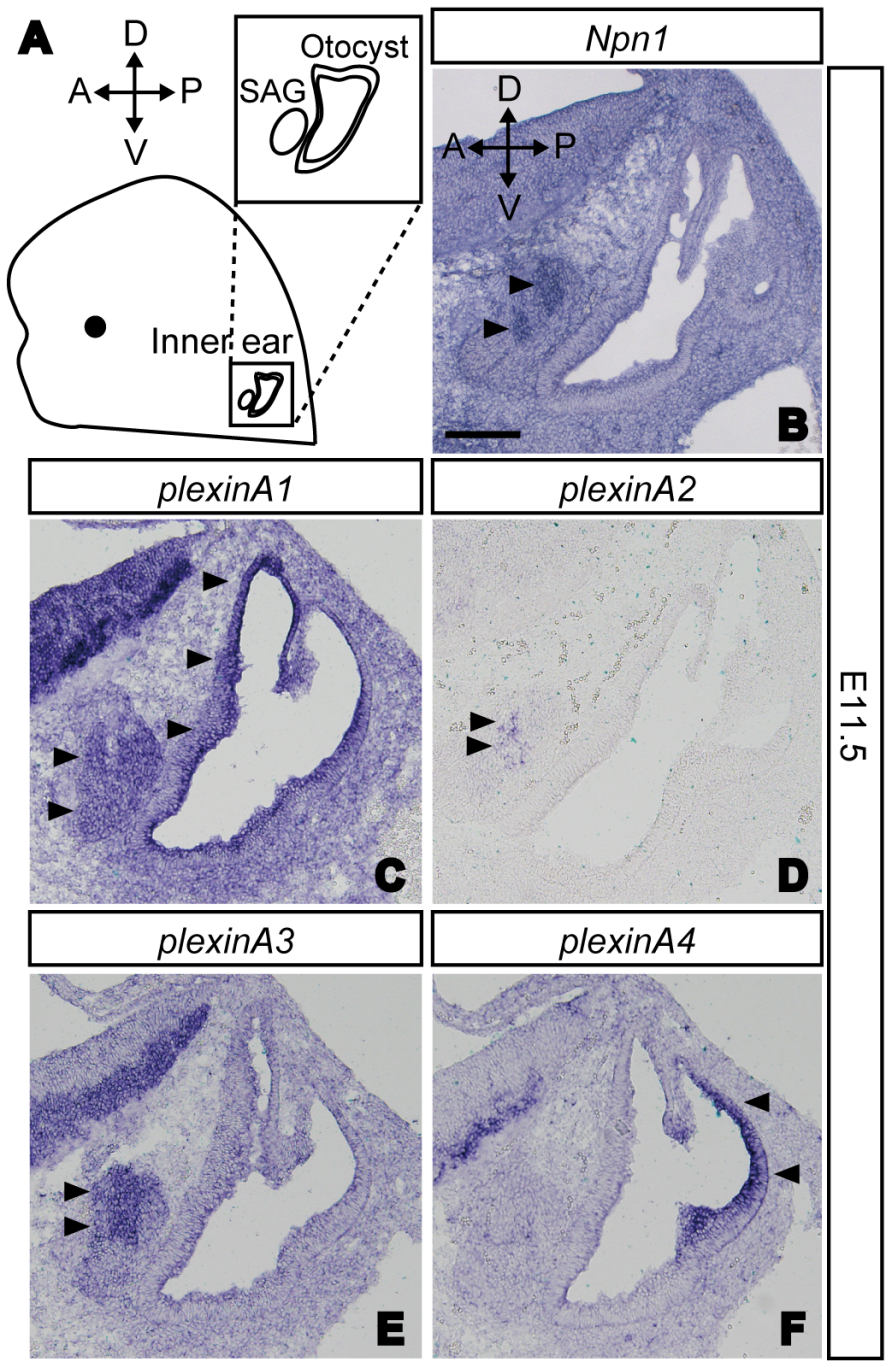
Expression patterns of *Npn1* and *plexinAs* in the developing inner ear at E11.5. (A) A schematic diagram of a typical mouse embryo showing the orientation of the SAG and the otocyst. (B) Expression of *Npn1* was found in SAG neurons (arrowheads). (C) Expression of *plexinA1* was found in SAG neurons and the otocyst (arrowheads). (D, E) *PlexinA2* and *plexinA3* were expressed in SAG neurons only (arrowheads). (F) *PlexinA4* transcripts were detected in the posterior part of the otocyst (arrowheads). Scale bar, 100 µm.

**Figure 2:**
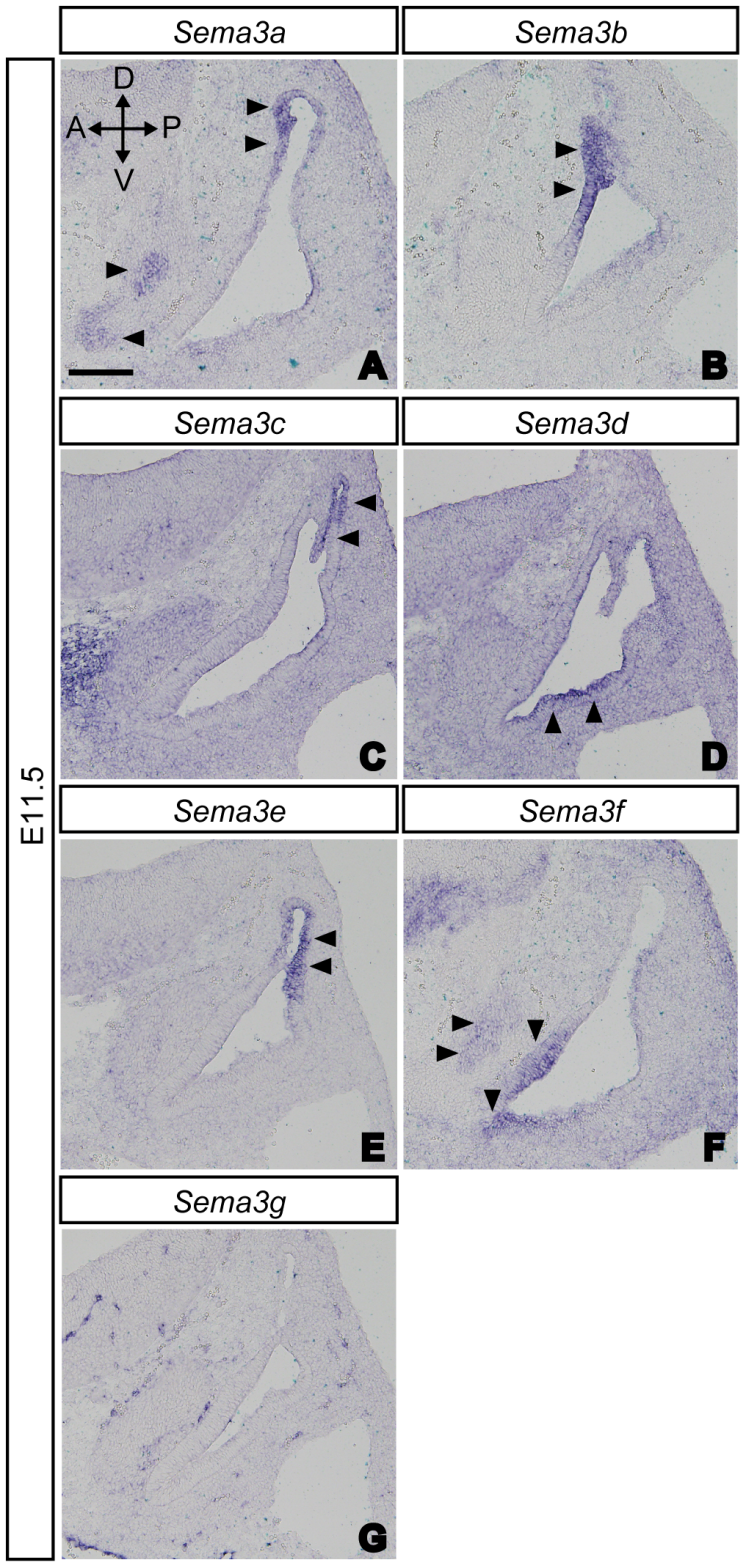
Expression patterns of *Sema3s* in the developing inner ear at E11.5. (A–C, E) Expression of *Sema3a*, *Sema3b*, *Sema3c* and *Sema3e* were found in the dorsal part of the otocyst (arrowheads). (D, F) Expression of *Sema3d* and *Sema3f* were found in the ventral part of the otocyst (arrowheads). (G) *Sema3g* transcripts were not found in either the otocyst or SAG neurons. (A, F) Weak expression of *Sema3a* and *Sema3f* were found in SAG neurons (arrowheads). Scale bar, 100 µm.

We first examined the expression patterns of Npn1, plexinAs and class 3 semaphorins in the inner ear of E11.5 wild-type mice by *in situ* hybridization. At E11.5, SAG afferents begin to navigate to their target sensory primordial [1], [3]. Previous studies that examined *plexinA* expression patterns in the developing inner ear focused only on E16.5 mouse embryos [13], [21] in which vestibular innervations had already developed. Detailed expression patterns of class 3 semaphorins, except for *Sema3e* in the developing inner ear [12], have yet to be examined in mouse embryos. In this study, we found that *plexinA1* expression was detected in both SAG neurons and the otocyst in E11.5 embryos (Fig. 1C). In addition, weak expression of *plexinA2* was found in only a subset of SAG neurons (Fig. 1D), while the expression of *Npn1* and *plexinA3* was found in dorsally localized SAG neurons (Fig. 1B, 1E). In contrast, strong expression of *plexinA4* was detected in the posterior portion of the otocyst (Fig. 1F). These data suggest that both plexinA1 and plexinA3 are potential coreceptors for Npn1 that may help to regulate the afferent projections of SAG neurons. Next we examined the expression of class 3 semaphorins in the inner ear. Expression of *Sema3a*, *Sema3b*, *Sema3c*, and *Sema3e* were found in the dorsal part of the otocyst at E11.5 (Fig. 2A–C, 2E). In contrast, *Sema3d* and *Sema3f* were found to be expressed in the ventral part of the otocyst (Fig. 2D, 2F). Weak expression of *Sema3a* and *Sema3f* was also found in SAG neurons (Fig. 2A, 2F). *Sema3g* was detected in vessel-like structures in the inner ear as well as in other organs and tissues, but not in the otocyst or SAG neurons (Fig. 2G). Based on these expression patterns, each class 3 semaphorin, with the exception of Sema3g, could act as a ligand for Npn1 in the inner ear.

### PlexinA1, plexinA3, and Sema3a are Required for the Proper Formation of Afferent Projections by SAG Neurons in the Inner Ear

**Figure 3:**
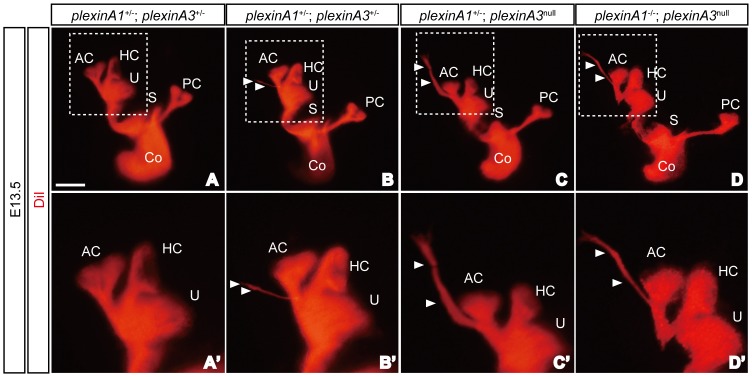
*PlexinA1* and *plexinA3* are required for proper sensory innervation of the inner ear. Some SAG neurons projected beyond the sensory patches and extended dorsally in E13.5 *plexinA1* ^+/−^; *plexinA3* ^+/−^ (A, B), *plexinA1* ^+/−^; *plexinA3* ^null^ (C) and *plexinA1* ^−/−^; *plexinA3* ^null^ (D) embryos (arrowheads). AC, anterior crista; Co, cochlea; HC, horizontal crista; PC, posterior crista; S, saccule; U, utricle. Scale bar, 500 µm.

A previous study using *Npn1* ^Sema*-*^ mice showed that Npn1 is required for the development of appropriate afferent SAG projections in the inner ear [11]. However, the relevant coreceptors or ligands for this Npn1 functioning remain unknown. Since *plexinA1* and *plexinA3* were strongly expressed by SAG neurons (Fig. 1C, 1E), we examined afferent projections in the inner ears of E13.5 *plexinA1* and *plexinA3* mutant embryos using DiI-labeling method [20]. Since *plexinA3* is localized in X chromosome, null mutants (*plexinA3* ^null^) were used to account for both *plexinA3* ^−/−^ and *plexinA3* ^−/y^ genotypes. We found that only a small portion (20–30%) of *plexinA1* ^−/−^ or *plexinA3* ^null^ embryos exhibited afferent projections of SAG neurons that extended beyond their normal target regions (Table 1). However, all *plexinA1* ^−/−^; *plexinA3* ^null^ double mutant embryos displayed afferent projections of SAG neurons that extended beyond the vestibular sensory patches (Fig. 3D and Table 1). Furthermore, embryos with several copies of *plexinA1* and *plexinA3* genes had aberrant projections that were thinner and shorter than those of *plexinA1* ^−/−^; *plexinA3* ^null^ embryos (Fig. 3B). This genetic evidence suggests that plexinA1 and plexinA3 have redundant roles in regulating afferent trajectories of SAG neurons in the developing inner ear, and that both plexinA1 and plexinA3 likely serve as coreceptors for Npn1.

**Table 1:** Incidence of phenotypes exhibiting overprojections of SAG neurons in *plexinA1*/*plexinA3*-knockout embryos at E13.5.

Genotype	Inner ears with aberrantneuronal projection
*plexinA1* ^+/+^; *plexinA3* ^+/−^	0% (0/3)
*plexinA1* ^+/−^; *plexinA3* ^intact^	0% (0/3)
*plexinA1* ^+/+^; *plexinA3* ^null^	27.3% (3/11)
*plexinA1* ^−/−^; *plexinA3* ^intact^	20% (1/5)
*plexinA1* ^+/−^; *plexinA3* ^+/−^	54.5% (6/11)
*plexinA1* ^+/−^; *plexinA3* ^null^	66.7% (2/3)
*plexinA1* ^−/−^; *plexinA3* ^+/−^	52.6% (10/19)
*plexinA1* ^−/−^; *plexinA3* ^null^	100% (6/6)

**Figure 4:**
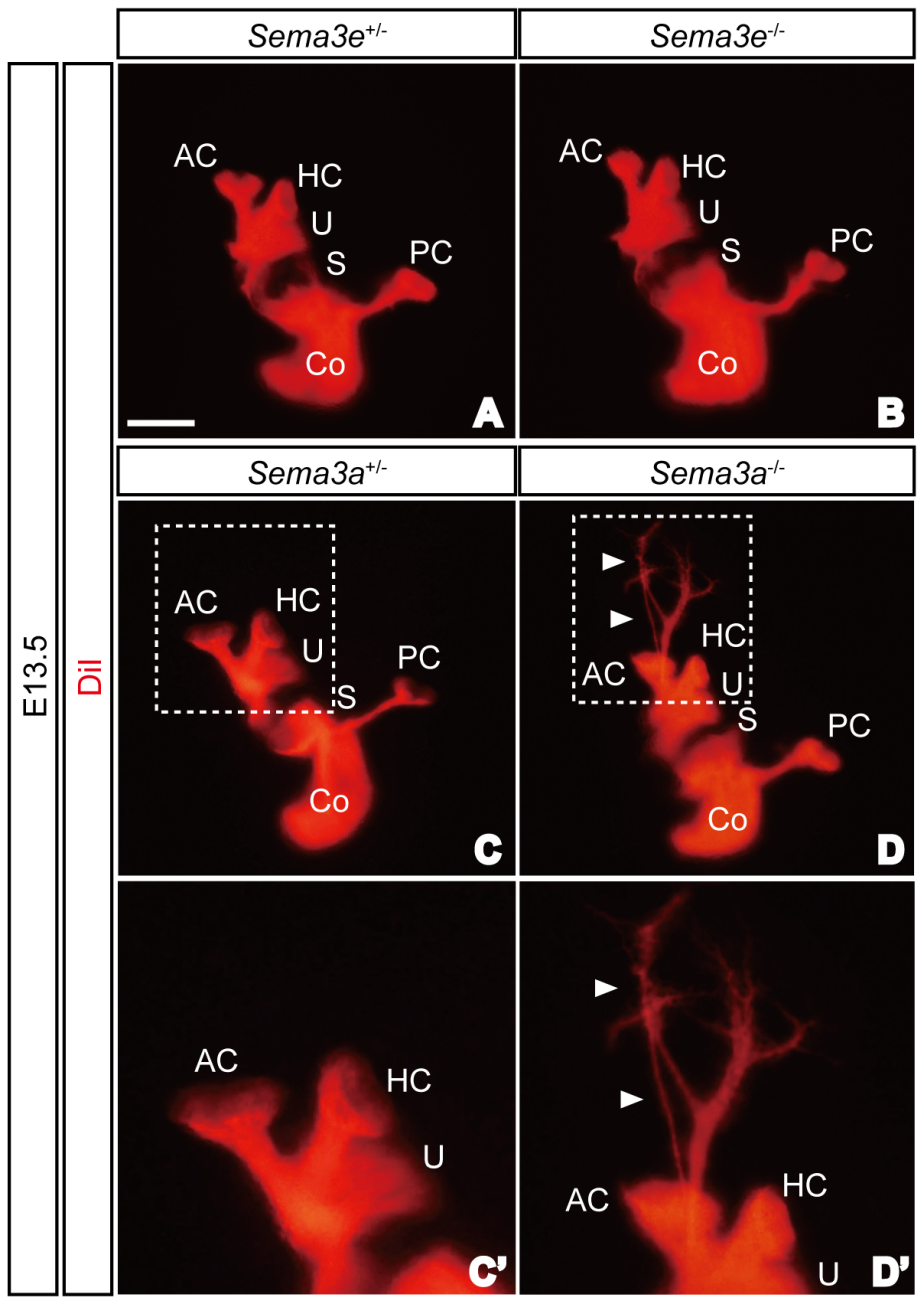
*Sema3a*, but not *Sema3e*, is required for proper sensory innervation of the inner ear. (A, B) Overall innervation patterns of *Sema3e* ^−/−^ embryos appeared normal at E13.5. (C, D) Some SAG neurons projected beyond the sensory patches and extended dorsally in *Sema3a* ^−/−^ embryos at E13.5 (arrowheads). AC, anterior crista; Co, cochlea; HC, horizontal crista; PC, posterior crista; S, saccule; U, utricle. Scale bar, 500 µm.

Next we examined the innervation patterns of SAG neurons in the inner ears of *Sema3a* and *Sema3e* mutant embryos since both *Sema3a* and *Sema3e* were strongly expressed in the otocyst (Fig. 2A, 2E). Despite strong expression of *Sema3e* in the dorsal otocyst in E11.5 embryos (Fig. 2E), the overall innervation patterns of SAG neurons in *Sema3e* ^−/−^ embryos appeared normal at E13.5 (Fig. 4A, 4B). This, however, does not exclude the possibility that other minor defects exist in *Sema3e* mutants. In contrast, some afferent projections of SAG neurons in *Sema3a* ^−/−^ embryos failed to stop at sensory epithelia and extended dorsally (Fig. 4C, 4D). All *Sema3a* ^−/−^ inner ears (6 out of 6) and 20% of *Sema3a* ^+/−^ inner ears (2 out of 10) showed similar defects in SAG afferent projections at E13.5. Thus Sema3a is required for the proper formation of sensory afferent projections in SAG neurons within the inner ear most likely functioning as a ligand for Npn1.

**Figure 5:**
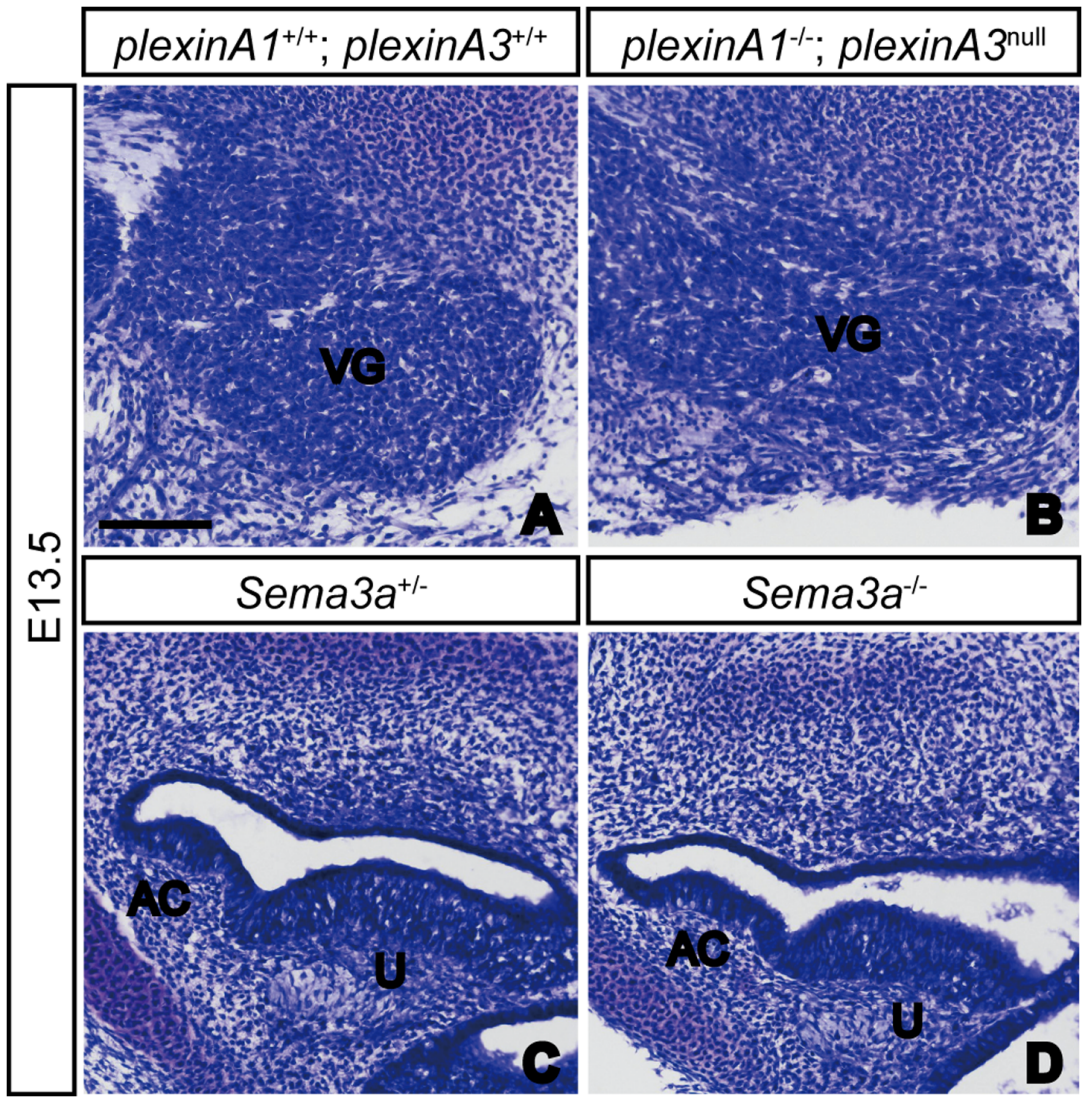
The vestibular ganglion and sensory epithelia appeared normal in *plexinA1*/*plexinA3* double mutant and *Sema3a* mutant embryos, respectively. In Nissl-stained sections of E13.5 embryos, no obvious differences were observed in the histology of vestibular ganglia between control and *plexinA1*/*plexinA3* double mutants (A, B). Furthermore, no obvious defects were detected in the morphology of anterior crista and utricular macula in *Sema3a* mutant embryos (C, D). AC, anterior crista; U, utricle; VG, vestibular ganglion. Scale bar, 100 µm.

Finally, it was still unclear whether plexinA1/plexinA3 and Sema3a exert direct control over SAG afferent projections, or whether this was a secondary effect arising from activities impacting the general development of sensory neurons and sensory epithelia, respectively. To clarify this issue, we performed a histological analysis of the morphology of the inner ears of both mutants, but found that sensory neurons in *plexinA1/plexinA3* double mutant embryos and sensory epithelia in *Sema3a* mutant embryos showed no obvious defects at E13.5 (Fig. 5 and data not shown). Although we cannot exclude the possibility that defects outside of the inner ear may affect the afferent projections of SAG neurons or that the loss of plexinA1, plexinA3 and Sema3a may affect the expression of other axon guidance molecules in the inner ear, our results suggest that these complexes of Sema3a, Npn1, and either plexinA1 or plexinA3 play some roles in controlling the trajectories of SAG neuron afferents in the developing inner ear.

## Discussion

In the inner ear, sensory afferent projections of SAG neurons need to be properly navigated to their final targets in order to form functional neural circuits for hearing and balance. A previous study using *Npn1* ^Sema*-*^ mice showed that Npn1 is required for the formation of sensory afferent projections in the inner ear [11]. However, several questions remained unanswered. Which plexins and semaphorins are coreceptors and ligands, respectively for Npn1 to regulate these afferent projections? In this study, we investigated the different possibilities.

Based on the strong expression of *plexinA1* and *plexinA3* by SAG neurons, we analyzed afferent projections of the SAG neurons of *plexinA1* single, *plexinA3* single, and *plexinA1/plexinA3* double mutant embryos. Similar to *Npn1* ^Sema*-*^ embryos [11], *plexinA1*/*plexinA3* double mutant embryos showed SAG neuron afferents that extended beyond the sensory patches and projected dorsally. These data suggest that plexinA1 and/or plexinA3 can be coreceptors for Npn1 that regulate the afferent trajectories of SAG neurons.

Our expression analysis also revealed that each class 3 semaphorin was expressed in different regions of the otocyst. Despite different expression patterns observed, our genetic evidence reveals that Sema3a is likely to be a functional ligand for Npn1, due to the similarities in defects in afferent projections of SAG neurons between *Sema3a* mutant embryos and *Npn1* ^Sema*-*^ embryos [11].

Class 3 semaphorins bind to Npn1 or Npn2, and the Npns make a receptor complex with plexinA family members to transduce their signals that control neural developmental events in the nervous system such as axon guidance. For example, the axons of the branchiomotor neurons are controlled by both Sema3a/Npn1/plexinA4 and Sema3f/Npn2/plexinA3 complexes during facial nerve development, whereas visceromotor neuron axons are controlled by Sema3a/Npn1/plexinA4 complex only [22]. In another scenario, complexes of Sema3a, Npn1 and either plexinA3 or plexinA4 help to regulate axonal projections of sensory neurons in the dorsal root ganglia [23]. In contrast to plexinA3 and plexinA4, plexinA1 and plexinA2 typically transduce their signals through interactions with class 6 semaphorins in the nervous system [14], [24]–[27].

Our genetic finding also suggests that Sema3a signaling can be transduced through Npn1/plexinA1 and/or Npn1/plexinA3 receptor complexes. However, an alternative possibility is that plexinA1 may function through its interaction with class 6 semaphorins, which would explain the stronger phenotype observed in *plexinA1/plexinA3* double mutants compared to the single mutants. Future studies will need to address the potential involvement of class 6 semaphorins in controlling sensory trajectories of SAG neurons.

Since class 3 semaphorins also bind to Npn2 [7]–[10], other class 3 semaphorins could potentially be ligands for Npn2 in the inner ear to exert control over sensory afferent projections through complexes with Npn2/plexinAs. Moreover, although we did not find any obvious defects in afferent projections of SAG neurons in *Sema3e* mutant embryos, there may be functional redundancies among other class 3 semaphorins. Thus, analysis of embryos of *Npn2* mutants as well as other *plexinA* and *Sema3* single or compound mutants will provide further molecular insights into how signaling by class 3 semaphorins regulates SAG sensory afferent projections.

Since deficient Npn1 signaling affected only a small portion of afferent projections of SAG neurons, different classes of semaphorin signaling molecules, as well as other guidance molecules, are likely involved in regulating sensory afferent trajectories in the inner ear. According to several databases such as the Allen Brain Atlas (http://www.brain-map.org/), the Auditory and Vestibular Gene Expression Database (http://goodrich.med.harvard.edu/), and the Eurexpress Transcriptome Atlas Database (http://www.eurexpress.org/), other classes of semaphorins and plexins are also expressed in the developing inner ear where they could potentially influence neural connectivities. Further detailed phenotypic analyses of mice lacking the various semaphorins and plexins, as well as other guidance molecules and receptors, will be necessary to fully elucidate the molecular basis of neural circuit formation in the inner ear.
